# Evaluating the Association of the Increase in the WIC Cash Value Benefit on the Diversity of MyPlate Fruits and Vegetables Redeemed and Consumed By Children in Low-Income Households

**DOI:** 10.1016/j.cdnut.2024.103778

**Published:** 2024-05-16

**Authors:** Alana M Chaney, Christopher E Anderson, Charles D Arnold, Shannon E Whaley, Lorrene D Ritchie, Gayathri R Pundi, Cassandra J Nguyen, Lauren E Au

**Affiliations:** 1 Department of Nutrition, University of California, Davis, CA, United States; 2 Research and Evaluation, Public Health Foundation Enterprises (PHFE) WIC, City of Industry, CA, United States; 3 Nutrition Policy Institute, Division of Agriculture and Natural Resources, University of California, Oakland, CA, United States; 4 Cooperative Extension, University of California, Davis, CA, United States

**Keywords:** The Special Supplemental Program for Women, Infants, and Children, WIC, cash value benefit, child fruit and vegetable intake, low-income households

## Abstract

**Background:**

Fruits and vegetables (FV) are a critical source of nutrients, yet children in the United States are not meeting the Dietary Guidelines for Americans (DGA). The monthly FV cash value benefit (CVB) included in the Special Supplemental Nutrition Program for Women, Infants, and Children (WIC)’s food package to support child FV intake (FVI) received a substantial increase for economic relief during the COVID-19 pandemic.

**Objectives:**

To evaluate how an expansion of the monthly WIC CVB to purchase FV for WIC children ages 1–4 y is associated with diversity in FV redeemed, and how changes in redeemed FV are related to FVI.

**Methods:**

Caregivers representing 1463 WIC-participating children recruited from Los Angeles County, California, completed surveys during the CVB augmentation (T1: CVB = $9/mo; T2 = $35/mo; T3 = $24/mo). Redeemed price look-up codes (PLUs), corresponding to a food item, were assigned to its corresponding MyPlate FV group. Multivariable generalized estimating equation regression models assessed changes in amount and diversity of FV redemption across MyPlate groups and associations between changes in FV diversity and changes in FVI.

**Results:**

Slightly over half of all households were food insecure (55%), half of the children were female (52%), and most were Hispanic (78%). Compared with T1, significant increases in the number of PLUs and dollars redeemed were observed in most MyPlate FV groups. From T1 to T2, significant increases in diversity scores were observed for total fruit (β: 1.6 pts; 95% confidence interval [CI]: 1.4, 1.7), total vegetable (β: 3.6 pts; 95%CI: 3.4, 3.9), and total FV (β:7.8 pts; 95%CI: 7.4, 8.2). Similarly, increases in diversity score were observed at T3 compared with T1. Changes in FV diversity redeemed were not associated with changes in FVI.

**Conclusions:**

During the CVB augmentation, WIC participants redeemed a greater amount and variety of FV according to DGA MyPlate recommendations, supporting its permanent increase.

## Introduction

Adequate diet quality in childhood is fundamental for maintaining physical health and establishing strong dietary habits that are likely to be tracked into adulthood [1,2]. Childhood is a period of rapid growth, and fruits and vegetables (FV) provide a significant source of nutrients for optimal growth [3,4]. Poor nutrition habits in young children have been associated with markers of obesity, dyslipidemia, and hypertension that persist throughout adulthood [[5], [6], [7]]. In addition to the amount of FV consumed, the diversity of FV selection has been positively associated with aspects of nutritional intake, such as increased vitamin and fiber consumption in adolescents [8]. Eating more types of FV has been associated with higher Healthy Eating Index-2010 (HEI-2010) scores in preschool-aged children from the 2005 to 2010 National Health and Nutrition Examination Survey [9]. In a sample of Texan 8th and 11th-graders, higher intake of fruit, vegetable, and combined FV diversity showed a similar pattern of higher HEI scores [10]. Furthermore, selecting a diversity of FV has been associated with eating a higher quantity of FV [11].

Although quantity and diversity of FV consumption plays an essential role in nutritional intake, 2- to 4-y-old children are not meeting recommendations set by the 2020–2025 Dietary Guidelines for Americans (DGAs) [12]. On average, children aged 2–4 y are eating <1 cup of total vegetables compared with the recommended 1–2 cups per day [12]. This shortfall from recommendations is even more pronounced for dark green, red, and orange vegetables [12]. Moreover, income and racial and ethnic disparities in fruit and vegetable intake (FVI) exist among youth. From 2015 to 2018, fruit and dark green vegetable consumption was significantly lower among children and adolescents aged 2–19 y in lower-income households in the United States [13]. In 2021, non-Hispanic (NH) Blacks had the highest percentage of children aged 1–5 y not meeting daily fruit (51%) and vegetable (65%) recommendations compared with NH White children not meeting daily fruit (26%) and vegetable (43%) recommendations [14]. Likewise, there were higher percentages of Asian children (fruit: 42%; vegetables: 48%) and Hispanic children (fruit: 34%; vegetable: 54%) also not meeting daily recommendations as compared with NH whites [14].

To address disparities in diet quality, the Special Supplemental Nutrition Program for Women, Infants, and Children (WIC) provides a food package and nutrition education that promotes FVI for low-income, nutritionally at-risk families [15]. The food package includes a monthly cash value benefit (CVB) that can be used to purchase fruits and vegetables, in addition to juice, milk, cereal, cheese, eggs, whole-wheat bread, fish, and legumes [16]. The CVB is unique from other items in the food package as it maximizes WIC participant autonomy, enabling households to purchase diverse selections of FV.

During the COVID-19 pandemic [17], a period that magnified food insecurity disparities, [[18], [19], [20], [21]] WIC increased the CVB for children aged 1–4 y from $9 per month to $35 per month in June 2021, which was revised to $24 in October 2021 [22,23]. A qualitative study of 51 WIC participants in Delaware showed that the increase in the CVB allotment allowed an increase in the amount and variety of FV redeemed [24]. WIC families’ perceptions of the CVB increase have also been positive, as they felt their children gained increased access to a variety of FV [[25], [26], [27]]. In low-income and racial and ethnically diverse households, a higher amount of CVB bolstered FV consumption, trips to the grocery store, and increased dietary variety [24]. At the increased amount, the CVB is aligned with National Academies of Sciences, Engineering, and Medicine recommendations, which ensures that the WIC CVB provides enough money to support 50% of the child FVI recommended in the DGAs [28]. Additionally, the USDA has recently announced that the WIC CVB will remain at the increased amount permanently to support its alignment with federal guidelines [29].

Previous studies have focused on qualitative results related to the CVB increase and perceived increases in FVI [[24], [25], [26], [27]]. However, very few studies have quantitatively examined how the increase in CVB influenced the variety of FV redeemed and consumed in children. Thus, the objective of this study is to evaluate whether the expansion of the monthly WIC CVB for the purchase of FV among children is associated with diversity in FV redeemed, and how the diversity of FV redeemed is related to child FVI in a California WIC sample. The hypothesis is that increasing the WIC CVB for a 1-y period will increase the diversity of FV redeemed, and the increase in diversity of FV redemption will be associated with increased FVI in children.

## Methods

### Participants

WIC participants in Los Angeles County, California, were invited to participate in a longitudinal study of a natural experiment. Caregivers with age-eligible children (1–4 y and 6 mo) were invited via text message to complete a 10-min online survey during April/May 2021 (T1: CVB = $9/mo). Families that completed that survey at baseline received a follow-up survey in September 2021 (T2: CVB = $35/mo) and a second follow-up survey in May 2022 (T3: CVB = $24/mo). Families included in the final cohort for this analysis must have completed the baseline survey, and at least one follow-up survey (*n* = 1578). The California Department of Health and Human Services Institutional Review Board approved the study.

### Child FVI

At each survey time point caregivers were asked to report children’s FVI using the National Health and Nutrition Examination Survey (NHANES) Dietary Screener Questionnaire (DSQ) [30]. Reported frequencies were used to estimate quantities of FVI (cups/day) using the publicly available National Cancer Institute–generated scoring algorithms by child age and gender. Total FVI included legumes, 100% fruit juice, and fried potatoes [31]. This method has been validated for use in studies with limited data [32] to evaluate child FVI [[33], [34], [35]].

### Redemption data

From June 2020 to June 2022, monthly CVB redemption data were collected on all participants. Redemption data are captured at the household level, meaning that families with >1 individual participating in WIC have a total CVB redemption amount combined from all WIC participants in that household. CVB redemption was averaged across 3 mo before each survey time point (T1: February–April 2021, T2: June–August 2021, and T3: February–April 2022). Therefore, this analysis only included participants who had redemption data 3 mo before each survey time point for a total sample of *n* = 1463. Redemption data were categorized by MyPlate groups [36] by sorting redeemed PLUs into berries, melons, other fruits, dark green vegetables, red/orange vegetables, beans/peas/lentils, starchy vegetables, and other vegetables (Supplemental Table 1). Using MyPlate, CVB redeemed was assessed by dollars (USD) redeemed, number of PLUs redeemed, and percent of monthly CVB redeemed.

### Diversity scores

The diversity scoring method used was adapted from Anderson et al. [37], which was developed encompassing concepts of disparity, variety, and balance [38]. Fruit, vegetable, and total FV diversity scores were calculated using the percent of CVB redeemed in each MyPlate group. MyPlate groups were used for fruits (consisting of 3 categories: berries, melons, and other fruits) and vegetables (consisting of 5 categories: dark green, starchy, beans/peas/lentils, red and orange, and other vegetables) equaling 8 total categories for FV [36] Assuming perfectly balanced redemption among groups, fruit redemption would be 33.3% in each group (i.e., 100/3 = 33.3%; and for vegetables 20% in each group, and for total fruits and vegetables 12.5% in each group). A fruit diversity score was calculated by first subtracting the percentage of CVB redemption in that commodity group from 33.3%. Then taking the absolute value of this difference and adding 10% (to ensure that the maximum contribution of any single category is truncated at 10 points). Truncation at a maximum of 10 points per MyPlate group was chosen to ensure that redemption in 1 MyPlate group would not overwhelm the variety of redemption observed in the other MyPlate groups. Then the inverse of this value was taken. Lastly, the overall fruit diversity score was determined by summing across all 3 fruit groups. The same process was repeated for the vegetable and FV scores using their perfect balanced redemption percentage in step 1 and summing across their total number of categories for overall diversity score (i.e., for vegetables using 20% in step 1 and summing across 5 groups). A higher diversity score indicates redemption in a greater number of groups and a greater balance of redemption among the different MyPlate groups.

### Covariates

Covariates included in regression models were selected based on previously reported associations with either redemption or FVI, or both, and which were thought to represent potential confounding factors. The demographics of the participants included variables such as race, age, and number of children within the household. Household food security was assessed within each survey using the validated USDA 6-item Food Security Survey module [39]. In previous studies on low-income children this tool has been shown to capture HFS for the 30 d before the survey [33].

### Statistical analysis

Children’s characteristics were summarized using frequencies and percentages for categorical variables. Continuous variables were described using means and standard deviations (SDs). Multivariable generalized estimating equation (GEE) regression models were used to test associations between the time period for the CVB augmentation and each redemption outcome, expressed as estimates and 95% confidence intervals (CIs; in My Plate groups for dollars, PLUs, and percentage and diversity scores).

Linear GEE models were also used to evaluate associations between change in diversity score and change in child intake (cups/day) (e.g., fruit diversity with fruit intake). In the original longitudinal cohort, increases in FVI were only observed among children not meeting FVI recommendations at baseline (<1.5 cups/d) [23]. Additional analyses were conducted to test for effect modification of the association between diversity scores and FVI by child baseline FVI at 3 levels: <1.5 cups/d, 1.5 to <2.5 cups/d, and ≥2.5 cups/d). Effect modification was also investigated for associations with number of children in the household, race and ethnicity, and HFS status. All models were adjusted for child race, gender, age at baseline, HFS status, and number of children in the household under age 18 y.

## Results

The majority of the children included in this study were Hispanic, with more Hispanic caregivers speaking primarily English (46% of the total sample) than Spanish (32% of the total sample) (Table 1). Approximately half of the children were female (52%) and the average age at baseline (T1) was 2.8 y. During the 3 CVB periods children had an average FVI of 2.3 cups/d (T1), 2.3 cups/d (T2), and 2.1 cups/d (T3), respectively (Supplemental Figure 1). Slightly more than half of all households were food insecure (55%), and the average number of children (younger than 18 y) in households was 2. During the 3 augmentation periods, households had an average CVB redemption of $7.4 (T1), $25.9 (T2), and $19.1 (T3), respectively.

**TABLE 1:** Characteristics of WIC-participating Southern California children included in the study

Characteristic	Full sample *n* = 1463
Number of children in household under the age of 18 y mean(SD)^1^	2.03 (0.79)
Number of WIC-participating children of age 1–4 y, mean (SD)^1^	1.18 (0.42)
Race/ethnicity of participating children, *n* (%)	
Asian	62 (4.24)
Black	145 (9.91)
Hispanic, English speaking	666 (45.52)
Hispanic, Spanish speaking	462 (31.58)
Other	90 (6.15)
White	38 (2.60)
Child gender, female, *n* (%)	765 (52.29)
Child age (y), mean (SD)	
Time period 1	2.75 (1.07)
Time period 2	3.16 (1.12)
Time period 3	3.40 (0.88)
Food secure^2^ households, *n* (%)^1^	591 (45.6)
Average CVB amount ($) redeemed by households each time period, mean (SD)^3^	
Time period 1: June 2020–May 2021 (9 USD/mo)	7.37 (4.59)
Time period 2: June 2021–September 2021 (35USD/mo)	25.89 (14.83)
Time period 3: October 2021–June 2022 (24 USD/mo)	19.06 (11.83)
Average fruit and vegetable intake^4^ (cups/d) by child each time period, mean (SD)	
Time period 1: April/May 2021	2.52 (0.90)
Time period 2: August/September 2021	2.47 (0.87)
Time period 3: April/May 2022	2.39 (0.80)

Abbreviations: CVB, cash value benefit; SD, standard deviation; USD, United States dollar; WIC, the Special Supplemental Nutrition Program for Women, Infants, and Children; PLU, price look-up codes.

1 Denominator *N* is 1297 households, smaller than the 1463 children, as households could have >1 child enrolled in the study.

2 Food security was captured using the USDA 6-item Food Security Survey module. Each affirmative response to the 6-items contributes 1-point; scores were dichotomized with those <2 indicating high/marginal household food security (HFS).

3 CVB redemption is captured at the household level not at the individual level, therefore CVB redemption value could exceed monthly CVB issued to each child.

4 Fruit and vegetable intake included legumes and included fried potatoes and 100% fruit juice.

Significant increases were observed in the number of PLUs redeemed for all MyPlate groups except for beans/peas/lentils when comparing T2 with T1, and T3 with T1 (Table 2). When comparing MyPlate groups at T2 with T1, the greatest increase of PLUs was found among other vegetables (β: 2.5 PLUs; 95%CI: 2.4, 2.6) followed by other fruits (β: 2.3 PLUs; 95%CI: 2.2, 2.4), and red/orange vegetables (β: 0.8 PLUs; 95%CI: 0.7, 0.8). Similarly, in T3 compared with T1, the 3 MyPlate groups with the greatest increase of PLUs redeemed were other vegetables (β: 1.6 PLUs; 95%CI: 1.5, 1.8), other fruits (β: 1.4 PLUs; 95%CI: 1.3, 1.5), and red/orange vegetables (β: 0.5 PLUs; 95%CI: 0.4, 0.5).

**TABLE 2:** Change in number of PLUs redeemed, dollars redeemed, and percentage of CVB redeemed in MyPlate fruit, and vegetable groups by WIC-participating households in Southern California during augmentation of the CVB for fruits and vegetables, June 2020–June 2022 (*n* = 1463)

	T1	T2	T3	T2 vs.T1	T3 vs. T1
	Number of PLU redeemed^1^				
	Mean (SD)			Association with CVB period β (95%CI)^2^	
Fruits					
Berries	0.14 (0.28)	0.37 (0.47)	0.33(0.43)	0.25 (0.22, 0.27)	0.20 (0.17, 0.23)
Melons	0.12 (0.25)	0.38 (0.46)	0.29 (0.40)	0.27 (0.25, 0.30)	0.17 (0.15, 0.20)
Other fruits	1.22 (0.97)	3.45 (2.15)	2.55 (1.68)	2.33 (2.23, 2.43)	1.37 (1.26, 1.48)
Vegetables					
Red/orange	0.39 (0.49)	1.13 (0.89)	0.90 (0.77)	0.75 (0.71, 0.80)	0.49 (0.44, 0.54)
Starchy	0.20 (0.34)	0.60 (0.63)	0.48 (0.54)	0.41 (0.37, 0.44)	0.27 (0.23, 0.31)
Dark green	0.14 (0.28)	0.43 (0.52)	0.32 (0.44)	0.31 (0.28, 0.33)	0.18 (0.15,0.21)
Other vegetables	1.08 (1.15)	3.51 (2.53)	2.69 (2.08)	2.51 (2.39, 2.63)	1.63 (1.50, 1.76)
Beans/legumes	0.00 (0.03)	0.01 (0.05)	0.01 (0.05)	0.003 (−0.000, 0.001)	0.002 (−0.002, 0.006)
	Dollars ($) redeemed^3^				
	Mean (SD)			Association with CVB period β (95%CI)^2^	
Fruits					
Berries	0.48 (1.10)	1.51 (2.37)	1.21 (1.88)	1.16 (1.02, 1.29)	0.81 (0.67, 0.95)
Melons	0.46 (1.04)	1.63 (2.63)	1.40 (2.25)	1.27 (1.11, 1.42)	1.00 (0.84, 1.16)
Other fruits	3.16 (2.80)	10.54 (7.42)	7.69 (6.12)	7.88 (7.51, 8.25)	4.76 (4.36, 5.17)
Vegetables					
Red/orange	0.70 (1.04)	2.63 (2.52)	1.77 (1.95)	1.99 (1.87, 2.12)	0.99 (0.87, 1.11)
Starchy	0.44 (0.84)	1.47 (1.79)	1.30 (1.74)	1.07 (0.98, 1.17)	0.82 (0.71, 0.94)
Dark green	0.30 (0.70)	1.08 (1.68)	0.63 (1.05)	0.85 (0.75, 0.94)	0.34 (0.26, 0.41)
Other vegetables	1.81 (2.00)	7.00 (5.60)	5.05 (4.23)	5.43 (5.14, 5.72)	3.24 (2.98, 3.51)
Beans/legumes	0.01 (0.11)	0.03 (0.40)	0.02 (0.22)	0.028 (0.002, 0.055)	0.010 (-0.007, 0.026)
	Percent of CVB dollars redeemed^4^				
	Mean (%), (SD)			Association with CVB period β (95%CI)^2^	
Fruits					
Berries	5.73 (13.34)	5.54 (9.12)	6.59 (11.35)	−0.08 (−0.82, 0.66)	1.15 (0.18, 2.11)
Melons	5.45 (12.74)	6.04 (9.91)	6.23 (10.20)	0.67 (−0.12, 1.46)	0.76 (−0.11, 1.64)
Other fruits	39.52 (29.78)	38.92 (19.99)	38.45 (21.53)	−0.66 (−2.23, 0.92)	0.93 (−2.79, 0.92)
Vegetables					
Red/orange	8.72 (12.95)	9.57 (9.31)	8.88 (10.09)	0.72 (−0.01, 1.44)	−0.38 (−1.25, 0.49)
Starchy	5.29 (10.82)	5.34 (6.61)	6.48 (9.15)	0.15 (−0.46, 0.76)	0.96 (0.15, 1.77)
Dark green	3.89 (9.94)	4.32 (7.70)	3.74 (7.71)	0.39 (−0.21, 0.99)	−0.23 (−0.90, 0.45)
Other vegetables	22.46 (22.65)	25.66 (16.26)	25.98 (17.82)	2.72 (1.51, 3.93)	2.92 (1.44, 4.40)
Beans/legumes	0.14 (2.01)	0.06 (0.74)	0.12 (1.30)	−0.05 (−0.15, 0.05)	−0.02 (−0.16, 0.11)
	Mean (SD)			Association with CVB period β (95%CI)^2^	
Total fruit diversity score^5^	3.20 (1.89)	4.74 (2.06)	4.38 (2.02)	1.55 (1.43, 1.67)	1.21(1.06, 1.36)
Total vegetable diversity score^6^	6.52 (4.16)	10.10 (3.90)	9.28 (3.87)	3.62 (3.39, 3.85)	2.77 (2.48, 3.06)
Total FV diversity score^7^	14.83 (7.52)	22.55 (7.93)	20.76 (7.36)	7.82 (7.40, 8.24)	6.02 (5.50, 6.55)

1 PLU redemption is expressed as the mean (standard deviation) number of PLUs redeemed per family in the specified category.

2 Estimate (95% confidence interval) for each redemption outcome was determined for T2 (35 USD/mo) and T3 (24 USD/mo) compared with T1 (9 USD/mo) in generalized estimating equations linear regression models adjusted for child race/ethnicity, gender, and age; household food insecurity and the number of household members under age 18 y. Models also accommodated clustering of observations within participating children and families.

3 Dollar amount is expressed as the mean (standard deviation) amount of cash value benefit (CVB) redeemed per family (USD) in the specified category during the 3 CVB amounts issued (T1: 9 USD/mo; T2: 35 USD/mo; T3: 24 USD/mo) during the study period.

4 Percentage of CVB is expressed as the percentage of 3 prior months where there was any redemption in the specified category during the study period.

5 Fruit diversity was captured as the sum (across all 3 MyPlate fruit groups) of the inverse of the absolute value of 33.3 minus the percent of redemption of fruits plus 10 [i.e., 1/(absolute value (33.3 − percent in specific category)+10)].

6 Vegetable diversity was captured as the sum (across all 5 MyPlate vegetable groups) of the inverse of the absolute value of 20.0 minus the percent of redemption of vegetables plus 10 [i.e., 1/(absolute value(20.0 − percentage in specific category)+10)].

7 Total FV diversity score was captured as the sum (across all 8 MyPlate groups) of the inverse of the absolute value of 12.5 minus the percent of redemption of redemption of fruit and vegetables plus 10 [i.e., 1/(absolute value (12.5 − percentage in specific category)+10)].

The households spent significantly more money on all MyPlate groups in T2 and T3 when compared with T1, except for beans/peas/lentils in T3. In T2, the largest increases in dollars redeemed were observed in other fruits (β: $7.88; 95%CI: 7.51, 8.25), other vegetables (β: $5.43; 95%CI: 5.14, 5.72), red/orange vegetables (β: $1.99; 95%CI: 1.87, 2.12), melons (β: $1.27; 95%CI: 1.11, 1.42), berries (β: $1.16; 95%CI: 1.02, 1.29), and starchy vegetables (β: $1.07; 95%CI: 0.98, 1.17). In T3 compared with T1, the greatest increases in dollars redeemed were observed in other fruits (β: $4.76; 95%CI: 4.36, 5.17), other vegetables (β: $3.24; 95%CI: 2.98, 3.51), melons (β: $1.00; 95%CI: 0.84, 1.16), red/orange vegetables (β: $0.99; 95%CI: 0.87, 1.11), and starchy vegetables (β: $0.82; 95%CI: 0.67, 0.95). In T2 compared with T1, a significantly greater percentage of monthly CVB dollars was only observed among other vegetables (β: 2.7%; 95%CI: 1.5, 3.9). In T3 compared with T1, significant increases in percentage of CVB dollars redeemed were observed among other vegetables (β: 2.9%; 95%CI: 1.4, 4.4), berries (β: 1.2%; 95%CI: 0.2, 2.1), and starchy vegetables (β: 1.0%; 95%CI: 0.2, 1.8).

Comparing T2 with T1, significant increases in percentage of CVB redeemed were observed for all 3 diversity scores: total fruit (β: 1.6 pts; 95%CI: 1.4, 1.7), total vegetable (β: 3.6 pts; 95%CI: 3.4, 3.9), and total FV (β: 7.8 pts; 95%CI: 7.4, 8.2). Similarly, significant increases were also observed in the diversity of CVB redeemed when comparing T3 with T1: total fruit (β: 1.2 pts; 95%CI: 1.1, 1.4), total vegetable (β: 2.8 pts; 95%CI: 2.5, 3.1), and total FV (β: 6.0 pts; 95%CI: 5.5, 6.6). No significant associations were observed between changes in diversity scores and child FVI (Table 3). Additionally, no effect modification by baseline FVI was observed for associations of changes in diversity scores and changes in FVI (Supplemental Table 2). Other variables evaluated as potential effect modifiers (food insecurity, race and ethnicity, and number of children in the household) did not significantly modify the association between change in diversity and change in FVI (data not shown).

**TABLE 3:** Adjusted association of change in diversity of fruit and vegetable score on fruit and vegetable intake in WIC-participating households in Southern California during augmentation of the CVB for fruits and vegetables (*n* = 1173)^1^

Association with change in child dietary intake^2^						
Change in diversity score^3^	Fruit intake, cups/d		Vegetable intake^4^, cups/d		Fruit and vegetable intake^4^, cups/d	
	β (95%CI)	*P*-value	β (95%CI)	*P*-value	β (95%CI)	*P*-value
**Total fruit diversity score**						
Time period 2 vs. time period 1	0.010 (−0.006, 0.030)	0.195	−0.003 (−0.01, 0.007)	0.584	0.007 (−0.016, 0.031)	0.556
Time period 3 vs. time period 1	−0.005 (−0.025, 0.015)	0.638	−0.002 (−0.014, 0.011)	0.814	−0.009 (−0.036, 0.017)	0.496
**Total vegetable diversity score**						
Time period 2 vs. time period 1	−0.002 (−0.011, 0.008)	0.722	0.001 (−0.005, 0.008)	0.701	0.000 (−0.013, 0.013)	0.995
Time period 3 vs. time period 1	0.001 (−0.009, 0.012)	0.805	0.005 (−0.001, 0.011)	0.121	0.005 (−0.008, 0.019)	0.422
**Total FV diversity score**						
Time period 2 vs. time period 1	0.001 (−0.004, 0.007)	0.629	−0.000 (−0.003, 0.003)	0.989	0.001 (−0.006, 0.008)	0.736
Time period 3 vs. time period 1	−0.000 (−0.006, 0.006)	0.951	0.002 (−0.002, 0.005)	0.307	0.001 (−0.007, 0.008)	0.813

Abbreviations: CVB, cash value benefit; USD, United States dollars; SD, standard deviation; WIC, the Special Supplemental Nutrition Program for Women, Infants, and Children; FV, fruit and vegetables.

1 Sample size ranges from 727–1,173 due to the number of completed surveys at each timepoint.

2 Estimate (95% confidence interval) for child dietary intake associations was determined for T2 (35 USD/mo) and T3 (24 USD/mo) compared to T1 (9 USD/mo) in generalized estimating equations linear regression adjusted for child race/ethnicity, sex, and age; household food insecurity and the number of household members under age 18; Models also accommodated clustering of observations within participating children and families.

3 Change in diversity was calculated as fruit, vegetable, and FV diversity score at time period 2 minus time period 1, and time period 3 minus time period 1, respectively.

4 Vegetable intake and FV intake included legumes, fried potatoes, and 100% fruit juice.

## Discussion

In this longitudinal study of critical public health investment, WIC-participating households purchased greater amounts and diversity of MyPlate FV during the increased CVB. Additionally, households were able to purchase more MyPlate vegetables such as red/orange, other, and starchy vegetables (e.g., corn, yams, green lima beans) that children are currently underconsuming [12]. Consuming a greater variety of FV is essential, especially during childhood as it has been associated with increased diet quality [9], meeting nutrient needs, and reducing the risk of diet-related diseases [40]. These findings are noteworthy as this study was conducted within a low-income population who often purchase foods of lower nutritional quality [41] and consume lower amounts [42] and variety [43] of FV. Furthermore, current literature shows that dietary habits established early in childhood persist throughout the lifespan [2,44,45]. As research has shown that utilizing MyPlate can improve diet quality [46,47] understanding the redemption of MyPlate food groups may support WIC staff in encouraging participants to consume FV in alignment with dietary guidance.

Significant increases for number of PLUs and dollars redeemed were observed in most of the MyPlate groups at T2 and T3 when comparing with T1. This is consistent with previous research as WIC-participating households redeemed significantly more money for the majority of FV PLU-group PLUs during the CVB augmentation [37]. The lack of change among percentage of CVB redeemed among foods has also been documented in previous literature [37]. Beans, peas, and lentils were the only MyPlate group with no consistent significant changes in the number of PLUs and the number of dollars redeemed from T3 compared with T1, with the monetary difference being very small at ∼$0.01. This nonsignificant difference may be due to beans being provided in the CA WIC package separately from the CVB [48]. Households also purchased a greater diversity of FV, as total fruit, vegetable, and FV diversity scores increased in both T2 and T3 compared with T1. This was also observed in previous studies where WIC participants expressed how they were able to redeem a greater variety of FV for their families due to the increased CVB amounts [24,27].

This study is unique as it investigates the variety of FV redeemed during the CVB increase and its association with child FVI. This analysis found no significant associations of FV variety redeemed with child FVI. In the study by Whaley et al. [23] using a similar population, FVI increased during the CVB augmentation among children with lower baseline FVI, and the greatest increase was observed among children with a baseline <1.5 cups/d. However, in this study, no association was observed between change in FV diversity redeemed with any diversity score (e.g., fruit, vegetable, or FV) from baseline and change in FVI from baseline at either time point (T2 or T3). This diversity of findings suggests that increases in diversity of purchases, rather than total purchases, may have differential associations with FVI. The augmented CVB value is estimated to only support ∼50% of a child’s FV recommendation [28], and therefore changes to diversity of purchases may not be of sufficient magnitude to detect differences in child FVI. Additionally, findings may reflect a spillover effect where other members of the household that are non-WIC participants may benefit by also consuming the increased diversity of the FV redeemed [49,50].

Strengths of this study include a large sample size with detailed redemption data down to the level of PLUs, allowing examination of the specific types of FV redeemed. Furthermore, the diverse population of Southern California allows the opportunity to understand how WIC changes impacted households from several racial and ethnic backgrounds. However, limitations within this study also exist. This study does not account for participation in the federal Supplemental Nutrition Assistance Program (SNAP), in which approximately one-third of WIC participants are also enrolled. Participating in multiple federal programs has been shown to improve food security [51], which is likely to work in combination with the WIC CVB to increase FVI. As the NHANES DSQ assessed child FVI, FVI could not be assessed in MyPlate groups although redemption was assessed at this level. Future studies using different methods of capturing FVI should investigate how CVB can impact the consumption of individual foods. Additionally, as FV diversity scores in this study represented the balance of MyPlate group redemption, this does not necessarily indicate better diet quality. Although a perfect balance of MyPlate groups is not needed for nutritional adequate diet, the DGA recommends consuming a variety of FV subgroups [40]. Vegetable intake is shown to decrease as children age [40], however, this study was unable to account for this by control group comparison because the CVB augmentation was implemented nationally. Lastly, the results of this study may not be generalizable to other states, as California has higher FVI than national FVI levels [52,53], emphasizing the need for additional research from a nationally representative sample.

In conclusion, WIC participants redeemed a greater amount and variety of FV during the CVB augmentation, adding to the evidence that supported the need for the increased CVB amount. Although further research is needed to understand how these changes impact child FVI among WIC participants nationwide, this study demonstrates that participating households utilized additional FV benefits for a broad diversity of FV, potentially improving nutritional outcomes. Lastly, this participant-focused change helped to address nutritional gaps that were highlighted during the COVID-19 pandemic, strengthening the WIC program.

## Author contributions

The authors’ responsibilities were as follows – AMC, CEA, LEA: designed research; AMC, CEA: conducted research; AMC, CEA, CDA: analyzed data; GRP, AMC: wrote the article; AMC: had primary responsibility for the final content; and all authors: read and approved the final manuscript.

## Conflict of interest

The authors report no conflicts of interest.

## Funding

Research reported in this publication was supported by the University of California Davis Public Impact Research Initiative, US Department of Agriculture/National Institute of Food and Agriculture Hatch Project# CA-D-NTR-2689-H, Healthy Eating Research, a national program of the Robert Wood Johnson Foundation, round 12, grant number 77239, and the David and Lucile Packard Foundation, grant number 2020-70267 and the University of California Office of the President Historically Black Colleges and Universities Fellowship. The content is solely the responsibility of the authors. It does not necessarily represent the official views of the United States Department of Agriculture or the University of California Office of the President.

## Data availability

The data described in the manuscript will not be made available because the data are confidential administrative data of the WIC program. The code book and analytic code will be made available upon request.
