# Supplementary material for: Evaluating the Association of the Increase in the WIC Cash Value Benefit on the Diversity of MyPlate Fruits and Vegetables Redeemed and Consumed By Children in Low-Income Households
Source: Curr Dev Nutr. 2024 May 16;8(6):103778. doi: 10.1016/j.cdnut.2024.103778 (PMC11215201; doi:10.1016/j.cdnut.2024.103778)
Supplement: Multimedia component 1 [file mmc1.docx]

| **Supplemental Table 1.** Price look-up Codes (PLUs) by MyPlate Categories redeemed in WIC-participating households in Southern California during augmentation of the CVB for fruits and vegetables | | |
| --- | --- | --- |
| My Plate Category | Description | PLU (Description) |
| 1 | Other fruit | 3002 (Apples Cantared), 3003 (Apples D'Estivale), 3005 (Apple GoldnDelcious Blush), 3006 (Apples Ingrid Marie), 3007 (Apples Lochbuie), 3008 (Apples Rubinette), 3010 (Apples Cripps Red), 3011 (Apples Worcester), 3015 (Pears Clara Friis), 3016 (Pears Concorde), 3027 (Oranges Shamouti), 3028 (Orange Delta Seedlss), 3029 (Tang/Mand Satsuma), 3030 (Tang/Mand Nova), 3031 (Tang/Mand JamacnTang), 3032 (Tang/Mand Ellendale), 3035 (Nectarne Whtflsh-TreeRpnd), 3037 (Pineapple Queen), 3038 (Passion Fruit Granadilla), 3040 (Pitahaya Red-Skin), 3041 (Rambutan), 3042 (Mangosteen), 3044 (Apricots Black), 3065 (Apples Cameo), 3071 (Apples Granny Smith), 3072 (Apples Lady), 3093 (Grapes Retailer Assigned), 3107 (Oranges Navel), 3108 (Oranges Valencia), 3110 (Oranges Navel), 3111 (Papaya/Pawpaw Red flsh), 3112 (Papaya/Pawpaw Meridol), 3113 (Peach-Flat White Flesh), 3114 (Mango Green), 3115 (Peaches Flat Yellow Flesh), 3117 (Peaches Yellow Flesh), 3118 (Pears Starkrimson), 3127 (Pomegranate), 3129 (Grapefruit Pummelo), 3144 (Tang/Mand FallGlo), 3155 (Oranges Midknight), 3157 (Grapefruit White), 3278 (Plumcot), 3279 (Kiwifruit Golden), 3280 (Kiwifruit Regular), 3283 (Apples Honeycrisp), 3284 (Apples Red Delicious), 3285 (Apples Golden Delicious), 3293 (Apples Scifresh), 3294 (Apples Scifresh), 3301 (Apples Cripps Red), 3302 (Apricots Regular), 3310 (Oranges Pera), 3313 (Peaches White Flesh), 3315 (Apples Scilate), 3319 (Pitahaya Yellow-Skin), 3357 (Cherries Reg/Red/Black), 3363 (Mango Bown&Kensgtnprd), 3372 (Orng Late Navel Vrty), 3374 (Oranges), 3380 (Pineapple Perola), 3381 (Soursop), 3382 (Sugar Apple), 3383 (Tang/Mand Clementine), 3388 (Tang/Mand Satsuma), 3389 (Tang/Mand Satsuma), 3420 (Pears Belle Du Jumet), 3422 (Apricots Interspecific), 3429 (Tang/Mand Hony/Mrctt), 3435 (Apples Pinova), 3437 (Nectarine Flat Yellow), 3438 (Apples Ambrosia), 3439 (Nectrine WhtFlsh Flat), 3440 (Pomegranate), 3449 (Grapes Sugrathirteen), 3450 (Grapes Sugranineteen), 3451 (Grapes Sugrathirtyfour), 3452 (Grapes Sugrathirtyfive), 3454 (Jackfruit Green), 3455 (Jackfruit Yellow), 3467 (Apples Regal 13-82), 3468 (Apples Honeycrisp), 3486 (Apples Cn121), 3487 (Apples Mn 55), 3488 (Mango Red), 3490 (APPLES MAIA 1), 3491 (GRAPES ARRA FIFTEEN), 3492 (GRAPES ARRA TWENTYNINE), 3496 (IFG Core Red Seedless Grap), 3498 (IFG Green Seedless Grapes), 3501 (IFG Novelty Green Seedless), 3507 (Cosmic Crisp Apple), 3510 (Ambrosia Apples), 3601 (Apples Huaguan), 3602 (Apples Belgica), 3603 (Apples Minneiska), 3604 (Apples Emmons), 3608 (Apples Sciros), 3610 (Plumcot-Intrspcfc Plm-Grn), 3613 (Apples Fuji Brak), 3616 (Apples Scilate), 3618 (Apples Opal), 3620 (Apples Plumac), 3627 (Apples Prema17), 3632 (Tang/Mand Dekopon), 4011 (Bananas Yellow), 4012 (Oranges Navel), 4013 (Oranges Navel), 4014 (Oranges Valencia), 4015 (Apples Red Delicious), 4016 (Apples Red Delicious), 4017 (Apples Granny Smith), 4019 (Apples Mcintosh), 4020 (Apples Golden Delicious), 4021 (Apples Golden Delicious), 4022 (Grape White/Green Seedlss), 4023 (Grapes Red Seedless), 4024 (Pear Bartlet/William/WBC), 4025 (Pears Anjou), 4026 (Pears Bosc/Beurre Bosc), 4027 (Grapefruit Ruby/Red/Pink), 4029 (Pineapple), 4030 (Kiwifruit Regular), 4033 (Lemons), 4035 (Nectarine Yellow Flesh), 4036 (Nectarine Yellow Flesh), 4037 (Peaches Yellow Flesh), 4038 (Peaches Yellow Flesh), 4039 (Plums Black), 4040 (Plums Black), 4041 (Plums Red), 4042 (Plums Red), 4043 (Peaches Yellow Flesh), 4044 (Peaches Yellow Flesh), 4045 (Cherries Reg/Red/Black), 4047 (Grapefruit Ruby/Red/Pink), 4048 (Limes Regular), 4051 (Mango Red), 4052 (Papaya/Pawpaw Regular), 4053 (Lemons), 4055 (Tangerines/Mandarins), 4056 (Grapes Blue/Black Seedlss), 4058 (Apples Haralson), 4096 (Apples Ginger Gold), 4098 (Apples Akane), 4101 (Apples Braeburn), 4103 (Apples Braeburn), 4106 (Apples Cortland), 4107 (Apples Crab), 4109 (Apples Crispin/Mutsu), 4122 (Apples Sciros), 4125 (Apples Empire), 4128 (Apples Cripps Pink), 4129 (Apples Fuji), 4130 (Apples Cripps Pink), 4131 (Apples Fuji), 4132 (Apples Gala), 4133 (Apples Gala), 4134 (Apples Gala), 4135 (Apples Gala), 4136 (Apples Golden Delicious), 4138 (Apples Granny Smith), 4139 (Apples Granny Smith), 4143 (Apples Jonamac), 4144 (Apples Jonagold), 4145 (Apples Jonagold), 4147 (Apples Jonagold), 4148 (Apples Jonathan), 4153 (Apples Mcintosh), 4156 (Apples Gravenstein), 4160 (Apples Pippin), 4162 (Apples Pippin), 4168 (Apples Red Delicious), 4170 (Apples Rome), 4173 (Apples Royal Gala), 4174 (Apples Royal Gala), 4186 (Bananas Yellow), 4188 (Nectrine Whtflsh-TreeRpn), 4191 (Apples Winesap), 4193 (Apples Retailer Assigned), 4194 (Apples Retailer Assigned), 4195 (Apples Retailer Assigned), 4196 (Apples Retailer Assigned), 4197 (Apples Retailer Assigned), 4201 (Apples Retailer Assigned), 4204 (Apples Retailer Assigned), 4205 (Apples Retailer Assigned), 4207 (Apples Retailer Assigned), 4210 (Apples Retailer Assigned), 4216 (Apples Retailer Assigned), 4218 (Apricots Regular), 4219 (Apricot Retailer Assigned), 4229 (Bananas Burro), 4230 (Bananas Dominique), 4233 (Bananas Apple/Manzano), 4234 (Bananas Baby/Nino), 4236 (Bananas Red), 4237 (Bananas Retailer Assigned), 4238 (Bananas Retailer Assigned), 4255 (Cactus Pear-Prickly Pear), 4256 (Carambola (Starfruit), 4257 (Cherimoya), 4258 (Cherries Gold/Rainier/Wht), 4259 (Cherries Retailer Assign), 4260 (Coconuts In Husk/Waternut), 4261 (Coconuts Husked), 4262 (Coconut Retailer Assign), 4264 (Dates Retailer Assigned), 4266 (Figs Black), 4267 (Figs Brown), 4269 (Figs Retailer Assigned), 4270 (Grapes Blue/Black Seeded), 4272 (Grapes Concord), 4273 (Grapes Red Seeded), 4274 (Grapes White/Green Seeded), 4275 (Grapes Retailer Assigned), 4276 (Grapes Retailer Assigned), 4277 (Grapes Retailer Assigned), 4279 (Grapefruit Pummelo), 4281 (Grapefruit Ruby/Red/Pink), 4282 (Grapefruit Ruby/Red/Pink), 4285 (Grapefruit Deep Red), 4286 (Grapefruit Deep Red), 4287 (Grapefruit Deep Red), 4288 (Grapefruit Deep Red), 4289 (Grapefruit Deep Red), 4294 (Grapefruit White), 4296 (Grapefruit RtlAssign), 4299 (Guava), 4301 (Kiwifruit Retailer Assign), 4303 (Kumquat), 4304 (Lemons Retailer Assigned), 4305 (Lime-Key-Inc.Mex&Wstindia), 4306 (Limes Retailer Assigned), 4307 (Longan), 4308 (Loquats), 4309 (Lychees), 4310 (Mamey), 4311 (Mango Green), 4312 (Mango Yellow), 4313 (Mango Retailer Assigned), 4314 (Mango Retailer Assigned), 4315 (Mango Retailer Assigned), 4316 (Mango Retailer Assigned), 4377 (Nectrine Yllwflsh-Treerpn), 4378 (Nectrine Yllwflsh-Treerpn), 4379 (Nectarine Retailer Assign), 4381 (Oranges Blood), 4382 (ORANGES JuiceORANGES Juice), 4383 (Tangelo Minneola), 4385 (Oranges Navel), 4388 (Oranges Valencia), 4389 (Oranges Retailer Assigned), 4392 (Oranges Retailer Assigned), 4394 (Papaya/Pawpaw Regular), 4395 (Papaya/Pawpaw Cking/Mex), 4397 (Passion Fruit Purple), 4400 (Peaches White Flesh), 4401 (Peaches White Flesh), 4402 (Peaches Yellow Flesh), 4404 (Peaches Retailer Assigned), 4406 (Pears Asian/Nashi), 4407 (Pears Asian/Nashi), 4408 (Pears Asian/Nashi), 4409 (Pear Bartlett/William/WBC), 4410 (Pears Bartlett), 4413 (Pears Bosc/Beurre Bosc), 4415 (Pears Red), 4416 (Pears Anjou), 4417 (Pears Anjou), 4418 (Pears Forelle/Corella), 4423 (Pears Tree Ripened), 4425 (Pears Retailer Assigned), 4426 (Pears Retailer Assigned), 4427 (Persimmon Reg-AmerPersim), 4428 (Persimmon/Kaki), 4430 (Pineapple), 4432 (Pineapple Jet Fresh), 4433 (Pineapple Retailer Assign), 4436 (Plums Italian Prune/Sugar), 4437 (Plums Purple), 4438 (Plums Purple), 4440 (Plums Tree Ripened), 4442 (Plums Yellow), 4443 (Plums Retailer Assigned), 4445 (Pomegranate), 4447 (Quince), 4448 (Tamarindo), 4449 (Tang/Mand Sunburst), 4450 (Tang/Mand Clementine), 4451 (Tang/Mand Dancy), 4452 (Tang/Mand Fairchild), 4453 (Tang/Mand Hony/Mrctt), 4454 (Tang/Mand Kinnow), 4455 (Tang/Mand Mandrin/Royal), 4456 (Tangelo), 4457 (Tang/Mand RetAssgn), 4459 (Tangelo Jamaican), 4471 (Other Fruit RetAssgn), 4472 (Other Fruit RetAssgn), 4473 (Other Fruit RetAssgn), 4474 (Other Fruit RetAssgn), 4477 (Other Fruit RetAssgn), 4478 (Other Fruit RetAssgn), 4480 (Other Fruit RetAssgn), 4482 (Other Fruit RetAssgn), 4485 (Other Fruit RetAssgn), 4486 (Other Fruit RetAssgn), 4492 (Grapefruit Ruby/Red/Pink), 4494 (Grapefruit Deep Red), 4495 (Grapefruit Deep Red), 4497 (Grape Sugraone/Atm Sdlss), 4498 (Grape White/Green Seedlss), 4499 (Grapes Crimson/Majestic), 4553 (Pears Taylors Gold), 4584 (Mango Green), 4635 (Grapes Red Seedless), 4636 (Grapes Red Globe), 4890 (Pears Chinese Yali), 4957 (Grapes Blue/Black Seeded), 4958 (Lemons), 4959 (Mango Red), 4961 (Mango Yellow), 93035 (Org Nectrn WhtFlshTreeRpn), 93047 (Org Dates Medjool), 93066 (Org Apples Cameo), 93093 (Org Grape Retailer Assign), 93107 (Org Oranges Navel), 93108 (Org Oranges Valencia), 93114 (Org Mango Green), 93283 (Org Apples Honeycrisp), 93382 (Org Sugar Apple), 93383 (Org Tang/Mand Clementine), 93468 (Org Apples Honeycrisp), 93618 (Org Apples Opal), 94011 (Org Bananas Yellow), 94012 (Org Oranges Navel), 94016 (Org Apples Red Delicious), 94017 (Org Apples Granny Smith), 94022 (Org Grape Wht/Grn Seedlss), 94023 (Org Grapes Red Seedless), 94024 (Org Pear Brtltt/Willm/WBC), 94025 (Org Pears Anjou), 94026 (Org Pears Bosc/Beurre), 94027 (Org Grapefrt Rby/Rd/Pink), 94030 (Org Kiwifruit Regular), 94033 (Org Lemons), 94035 (Org Nectarine YellwFlesh), 94036 (Org Nectarine Yell\WFlesh), 94037 (Org Peaches Yellow Flesh), 94039 (Org Plums Black), 94040 (Org Plums Black), 94042 (Org Plums Red), 94044 (Org Peaches YellowFlesh), 94047 (Org Grapefrt Rby/Rd/Pink), 94048 (Org Lime-Reg), 94053 (Org Lemons), 94056 (Org Grape Blu/Blk Seedlss), 94128 (Org Apples Cripps Pink), 94129 (Org Apples Fuji), 94130 (Org Apples Cripps Pink), 94131 (Org Apples Fuji), 94132 (Org Apples Gala), 94133 (Org Apples Gala), 94134 (Org Apples Gala), 94135 (Org Apples Gala), 94137 (Org Apple GldnDelicious), 94138 (Org Apples Granny Smith), 94139 (Org Apples Granny Smith), 94188 (Org Nectrn WhtflshTreeRpn), 94218 (Org Apricots Regular), 94288 (Org Grapefruit Deep Red), 94311 (Org Mango Green), 94312 (Org Mango Yellow), 94378 (Org Nectrn Ylwflsh-Treerp), 94382 (ORG ORANGES Juice), 94383 (Org Tangelo Minneola), 94388 (Org Oranges Valencia), 94400 (Org Peaches White Flesh), 94401 (Org Peaches White Flesh), 94409 (Org Pear Brtltt/Willm/WBC), 94413 (Org Pears Bosc/Beurre), 94416 (Org Pears Anjou), 94430 (Org Pineapple), 94457 (Org Tang/Mand RtlAssgn), 94497 (Org Grape Sgron/AutmSdlss), 94584 (Org Mango Green), 94635 (Org Grapes Red Seedless), 94958 (Org Lemons), 94959 (Org Mango Red) |
| 2 | Berries | 3039 (Phys/CapeGoosebryGrndCher), 3355 (Berries Strawberries), 4028 (Berries Strawberries), 4054 (Berries Raspberries), 4239 (Berries Blackberries), 4240 (Berries Blueberries), 4241 (Berries Boysenberries), 4242 (Berries Cranberries), 4246 (Berries Strawberries), 4247 (Berries Strawberries), 4248 (Berries Strawberries), 4250 (Berries Strawberries), 4252 (Berries Retailer Assigned), 4323 (Berries Strawberries), 93355 (Org Berries Strawberries), 94028 (Org Berries Strawberries), 94054 (Org Berries Raspberries), 94240 (Org Berries Blueberries), 94248 (Org Berries Strawberries) |
| 3 | Melon | 3100 (Melon Gold Honeydew), 3281 (Melon Watermelon), 3421 (Melon Watermelon), 3456 (Melon Winter), 3470 (Melon Watermelon), 3494 (MELON Watermelon - Yellow), 3623 (Melon Hami), 4031 (Melon Watermelon), 4032 (Melon Watermelon), 4034 (Melon Hnydew/Wht Honeydew), 4049 (Melon Cantaloup/Rockmelon), 4050 (Melon Cantaloup/Rockmelon), 4317 (Melon Canary/Yllw Honeydw), 4321 (Melon Cinnabar), 4322 (Melon Crenshaw), 4326 (Melon Galia), 4327 (Melon Orange Flsh/Cantlin), 4329 (Melon Hnydew/Wht Honeydew), 4332 (Melon Muskmelon), 4334 (Melon Persian), 4340 (Melon Watermelon), 4342 (Melon RetAssgn-Inc.Precut), 4343 (Melon RetAssgn-Inc.Precut), 4344 (Melon RetAssgn-Inc.Precut), 4346 (Melon RetAssgn-Inc.Precut), 4347 (Melon RetAssgn-Inc.Precut), 4352 (Melon RetAssgn-Inc.Precut), 4359 (Melon RetAssgn-Inc.Precut), 4363 (Melon RetAssgn-Inc.Precut), 4375 (Melon RetAssgn-Inc.Precut), 93100 (Org Melon Gold Honeydew), 93421 (Org Melon Watermelon), 94032 (Org Melon Watermelon), 94034 (Org Melon Hnydw/WhtHnydw), 94050 (Org Melon Cantaloup/Rkmln) |
| 4 | Other vegetable | 3051 (Cabbage SprCabbg/Spr Grns), 3055 (Pepper-Elongated), 3056 (Pepper-Elongated), 3058 (Pepper-Elongated), 3059 (Squash Crown Prince), 3089 (Eggplant-Aubergne-Chinese), 3103 (Mushrooms Enoki), 3119 (Peppr-Bell-Grnhse), 3120 (Peppr-Bell-Grnhse), 3141 (Squash Opo), 3273 (Beets Golden), 3286 (Onions Sweet Red Italian), 3327 (Lettc Mignon-SmRdButterhd), 3331 (Onions Red Fresh), 3392 (Asparagus Green), 3399 (Garlic Regular), 3401 (Garlic One-Clove Types), 3402 (Leeks Regular), 3412 (Onions Yellow/Brown Fresh), 3418 (Squash Zucchini/Courgette), 3436 (Cauliflower Orange), 3462 (Chives Garlic/Chinese), 3471 (Cactus Lf-Nopales-Bby), 4046 (Avocados Hass), 4061 (Lettuce Iceberg), 4062 (Cucumbr Green/Ridge/Short), 4065 (Peppr-Bell-FldGrwn), 4066 (Beans Green/French), 4067 (Squash Zucchini/Courgette), 4068 (Onion Grn-Scallion/Spring), 4069 (Cabbage Green), 4070 (Celery Bunch), 4071 (Celery Bunch), 4079 (Cauliflower), 4080 (Asparagus Green), 4081 (Eggplnt-Aubergine Regular), 4082 (Onions Red), 4084 (Artichokes), 4085 (Mushrooms Regular), 4086 (Squash Yellow Zuccini), 4089 (Radish Bunched Red), 4092 (Peas ChSnwPea/Pd/MngTout), 4093 (Onions Yellow/Brown), 4095 (Turnip Yellow), 4159 (Onions Vidalia), 4161 (Onions Texas Sweet), 4163 (Onions Walla Walla), 4165 (Onions California Sweet), 4166 (Onions Other Sweet), 4221 (Avocados Green), 4222 (Avocados Green), 4223 (Avocados Green), 4224 (Avocados Green), 4225 (Avocados Hass), 4226 (Avocado Cocktail/Seedless), 4227 (Avocado Retailer Assigned), 4228 (Avocado Retailer Assigned), 4500 (Oth Veg RetAssgn), 4501 (Oth Veg RetAssgn), 4502 (Oth Veg RetAssgn), 4503 (Oth Veg RetAssgn), 4504 (Oth Veg RetAssgn), 4505 (Oth Veg RetAssgn), 4506 (Oth Veg RetAssgn), 4508 (Oth Veg RetAssgn), 4509 (Oth Veg RetAssgn), 4512 (Oth Veg RetAssgn), 4513 (Oth Veg RetAssgn), 4514 (Alfalfa Sprouts), 4515 (Fennel-Florenc/Sweet/Bulb), 4516 (Artichokes), 4518 (Artichokes Purple), 4521 (Asparagus Green), 4522 (Asparagus White), 4524 (Asparagus Tips), 4525 (Asparagus Retailer Assign), 4527 (Beans Chinese Long/Snake), 4530 (Beans Pole/Runner/Stick), 4531 (Beans Purple Hull), 4533 (Beans Wax/Yellow), 4536 (Bean Sprout-MungBeanSprt), 4537 (Beets Baby Golden), 4538 (Beets Baby Red), 4539 (Beets Bunch), 4540 (Beets Loose), 4541 (Beets Retailer Assigned), 4550 (Brussels Sprouts), 4551 (Brussel Sprout RtlAssgn), 4552 (Cabbage Chnse/Napa/Wngbok), 4554 (Cabbage Red), 4555 (Cabbage Savoy Green), 4556 (Cabbage Retailer Assigned), 4557 (Cabbage Retailer Assigned), 4558 (Cactus Leaf-Nopales), 4566 (Cauliflower Florettes), 4567 (Cauliflower Green), 4568 (Cauliflower Purple), 4572 (Cauliflower), 4575 (Celery Hearts), 4576 (Celery Celery Sticks), 4577 (Celery Retailer Assigned), 4580 (Celery Retailer Assigned), 4582 (Celery Bunch), 4583 (Celery Bunch), 4585 (Celery Root/Celeriac), 4592 (Cucumber Armenian), 4593 (Cucumber English Seedless), 4594 (Cucumber Japanese/White), 4595 (Cucumber Lemon), 4596 (Cucumber Pickling/Gherkin), 4597 (Cucumber Retailer Assgn), 4598 (Daikon (See Also Radish), ), 4599 (Eggplant (Aubergine), Baby), 4600 (Eggplant-Aubrgin-BbyWht), 4601 (Eggplt-Aubergine-Japanese), 4603 (Eggplant-Aubrgin-RetAssgn), 4606 (Fiddlehead Ferns), 4608 (Garlic Regular), 4609 (Garlic Elephant), 4610 (Garlic Retailer Assigned), 4611 (Garlic Retailer Assigned), 4612 (Ginger Root Regular), 4613 (Gingerroot Retailr Assgn), 4628 (Kohlrabi), 4629 (Leeks Regular), 4641 (Lettuce Retailer Assigned), 4645 (Mushrooms Regular Button), 4648 (Mushrm Cremini/Brn/SwsBrn), 4649 (Mushrooms Oyster), 4650 (Mushrooms Portabella), 4651 (Mushrooms Shiitake), 4652 (Mushrooms Wood Ear), 4653 (Mushrooms Retailer Assign), 4654 (Mushrooms Retailer Assign), 4655 (Okra Regular (Green), ), 4656 (Okra Chinese), 4658 (Onions Boiling), 4659 (Onions Bulb), 4660 (Onions Pearl), 4662 (Onions Shallots), 4663 (Onions White), 4665 (Onions Yellow/Brown), 4666 (Onions Retailer Assigned), 4667 (Onions Retailer Assigned), 4668 (Onions Retailer Assigned), 4669 (Onions Retailer Assigned), 4670 (Onions Retailer Assigned), 4675 (Peas Sugar Snap), 4678 (Pepper-BananYlwLng), 4679 (Pepper-Bell-FldGrwn), 4680 (Pepper-Bell-FldGrwn), 4681 (Pepper-Bell-FldGrwn), 4686 (Pepper-Chili), 4687 (Pepper-Cubanelle), 4689 (Pepper-Bell-Grnhse), 4693 (Pepper-Jalapeno), 4696 (Pepper-Long Hot), 4701 (Pasilla-Green/Chilaca), 4705 (Pepper-Poblano), 4709 (Pepper-Serrano), 4710 (Pepper-RetAssgn), 4711 (Pepper-RetAssgn), 4712 (Pepper-RetAssgn), 4713 (Pepper-RetAssgn), 4714 (Pepper-RetAssgn), 4715 (Pepper-RetAssgn), 4716 (Pepper-RetAssgn), 4717 (Pepper-RetAssgn), 4719 (Pepper-RetAssgn), 4720 (Pepper-RetAssgn), 4721 (Pepper-RetAssgn), 4722 (Pepper-RetAssgn), 4738 (Radicchio), 4739 (Radish Black), 4740 (Radish Bunched White), 4742 (Radish Red), 4743 (Radish White/Icicle), 4744 (Radish Retailer Assigned), 4747 (Rutabagas (Swede), Reglr), 4748 (Rutabagas-Swede RetAssgn), 4756 (Squash Bbygrn Zucch/Crgtt), 4757 (Squash Banana), 4761 (Squash Chayote/Choko), 4762 (Artichokes), 4767 (Squash Golden Nugget), 4770 (Avocados Hass), 4771 (Avocados Green), 4773 (Squash Patty Pan/Summer), 4775 (Squash Scallopini), 4776 (Squash Spagh/VegSpaghetti), 4777 (Squash Sunburst (Yellow), ), 4781 (Squash White), 4782 (Squash Yellow), 4783 (Bitter Mln/Btr Grd-FooQua), 4784 (Squash Yellow), 4785 (Squash Retailer Assigned), 4786 (Squash Retailer Assigned), 4787 (Squash Retailer Assigned), 4788 (Squash Retailer Assigned), 4789 (Squash Retailer Assigned), 4801 (Tomatoes Tomatillos/Husk), 4811 (Turnip Purple Top), 4812 (Turnip White), 4820 (Oth Veg Retailer Assign), 4821 (Oth Veg Retailer Assign), 4822 (Oth Veg Retailer Assign), 4824 (Oth Veg Retailer Assign), 4826 (Oth Veg Retailer Assign), 4827 (Oth Veg Retailer Assign), 4828 (Oth Veg Retailer Assign), 4830 (Oth Veg Retailer Assign), 4831 (Oth Veg Retailer Assign), 4834 (Oth Veg Retailer Assign), 4835 (Oth Veg Retailer Assign), 4836 (Oth Veg Retailer Assign), 4837 (Oth Veg Retailer Assign), 4838 (Oth Veg Retailer Assign), 4841 (Oth Veg Retailer Assign), 4843 (Oth Veg Retailer Assign), 4844 (Oth Veg Retailer Assign), 4846 (Oth Veg Retailer Assign), 4847 (Oth Veg Retailer Assign), 4848 (Oth Veg Retailer Assign), 4849 (Oth Veg Retailer Assign), 4850 (Oth Veg Retailer Assign), 4853 (Oth Veg Retailer Assign), 4855 (Oth Veg Retailer Assign), 4856 (Oth Veg Retailer Assign), 4859 (Oth Veg Retailer Assign), 93121 (Org Pepper-BellGrnhs), 93273 (Org Beets Golden), 94046 (Org Avocados Hass), 94061 (Org Lettuce Iceberg), 94062 (Org Cucumbr Grn/Rdg/Shrt), 94065 (Org Peppr-Bell FldGrwn), 94067 (Org Squash Zucc/Crgt), 94068 (Org Onion Grn-Scalln/Spr), 94069 (Org Cabbage Green), 94070 (Org Celery Bunch), 94079 (Org Cauliflower), 94080 (Org Asparagus Green), 94081 (Org Eggplnt-Aubergine-Reg), 94082 (Org Onions Red), 94084 (Org Artichokes), 94085 (Org Mushrooms Regular), 94088 (Org Pepper-Bell-FldGrn), 94089 (Org Radishbunch-Red), 94093 (Org Onions Yellow/Brown), 94225 (Org Avocados Hass), 94227 (Org Avocado RetailAssign), 94514 (Org Alfalfa Sprouts), 94515 (Org Fennel Flrnc/Sw/Bulb), 94533 (Org Beans Wax/Yellow), 94539 (Org Beets Bunch), 94550 (Org Brussels Sprouts), 94554 (Org Cabbage Red), 94583 (Org Celery Bunch), 94593 (Org Cucumber English), 94596 (Org Cucmbr Pklng/Gherkin), 94597 (Org Cucmbr Ret.Assign), 94612 (Org Ginger Root Regular), 94613 (Org Gingerroot RetlrAssgn), 94629 (Org Leeks Regular), 94645 (Org Mushroom Reg-Button), 94663 (Org Onions White), 94680 (Org Pepper-FldGrwn), 94681 (Org Pepper-FldGrwn), 94682 (Org Pepper-FldGrwn), 94688 (Org Pepper-BellGrnhse), 94689 (Org Pepper-BellGrnhse), 94693 (Org Pepper-Jalapeno), 94750 (Org Squash Acorn/TblQueen), 94759 (Org Squash Butternut), 94769 (Org Squash Kabocha), 94776 (Org Squash Spaghetti/Veg), 94782 (Org Squash Yellow), 94784 (Org Squash Yellow) |
| 5 | Orange/red vegetables | 3054 (Pepper-Elongated), 3121 (Peppr-Bell-Grnhse), 3125 (Pepper-Habanero), 3132 (Pumpkin White), 3133 (Pumpkin White), 3134 (Pumpkin Pie Pumpkin), 3143 (Squash Acorn), 3145 (Tomato Plum/Itl/Salad/Rma), 3148 (Tomatoes Regular), 3149 (Tomatoes Regular), 3151 (Tomatoes Vine Ripe Reg), 3282 (Org TomtoVinePlmItlSldRma), 3288 (Sw Pot/Yam/Kumara-RetAssn), 3333 (Sw Potato/Yam/Kumara), 3334 (Sw Potato/Yam/Kumara), 3423 (Tomatoes Heirloom), 4063 (Tomatoes Regular), 4064 (Tomatoes Regular), 4074 (Sw Potat/Yam/Kumara), 4087 (Tomato Plum/Itl/Salad/Rma), 4088 (Peppr-Bell-FldGrwn), 4091 (Sw Potato/Yam/Kumara Whte), 4094 (Carrots Bunch), 4560 (Carrots Baby), 4561 (Carrots French), 4562 (Carrots Loose), 4563 (Carrots Carrot Sticks), 4564 (Carrots Retailer Assigned), 4565 (Carrots Retailer Assigned), 4664 (Tomatoes Regular), 4677 (Pepper-Anahm-Gr&Rd), 4682 (Pepper-Bell-FldGrwn), 4688 (Pepper-Bell-Grnhse), 4690 (Pepper-HungarnHot), 4692 (Pepper-Hungrn Wax), 4694 (Pepper-Capsicum-Jalapeno), 4697 (Pepper-Long Hot), 4700 (Pepper-NewMexico), 4702 (Pasilla-Red/Chilaca), 4706 (Pepper-Red Cheese), 4735 (Pumpkin Regular), 4736 (Pumpkin Retailr Assign), 4737 (Pumpkin Retailr Assign), 4750 (Squash Acorn/Table Queen), 4751 (Squash Acorn), 4752 (Squash Acorn), 4759 (Squash Butternut), 4760 (Squash Calabaza), 4768 (Squash Hubbard), 4769 (Squash Kabocha), 4772 (Pepper-Chili), 4778 (Tomatoes Regular), 4796 (Tomatoes Cherry), 4797 (Tomatoes Cherry), 4798 (Tomato Gnhse/Hydropnc/Reg), 4799 (Tomato Gnhse/Hydropnc/Reg), 4800 (Tomatoes Native/Hm Grwn), 4803 (Tomatoes Teardrop/Pear), 4804 (Tomatoes Teardrop/Pear), 4805 (Tomatoes Vine Ripe Reg), 4806 (Tomatoes Retailer Assign), 4807 (Tomatoes Retailer Assign), 4808 (Tomatoes Retailer Assign), 4816 (Sw Potat/Yam/Kumra Gold), 4817 (Sw Potat/Yam/Kumara), 93151 (Org Tomato Vine Ripe Reg), 93512 (ORG TOMATOES ROUND), 94087 (Org TomtoPlm/Itl/Sald/Rma), 94094 (Org Carrots Bunch), 94664 (Org Tomatoes Regular), 94796 (Org Tomatoes Cherry), 94805 (Org Tomato Vine Ripe Reg) |
| 6 | Starchy vegetable | 3287 (Bananas Hawaiian Plantain), 3414 (Potato Baking), 4072 (Potato Russet), 4073 (Potato Red), 4077 (Corn Sweet Corn White), 4078 (Corn Sweet Corn Yellow), 4083 (Potato White), 4231 (Bananas Green), 4235 (Bananas Plantain/Macho), 4254 (Breadfruit), 4546 (Boniato-Sweet Potato), 4589 (Corn Sweet Corn Baby), 4590 (Corn Sweet Corn Bi-Color), 4591 (Corn Retailer Assigned), 4625 (HORSERADISH ROOT), 4626 (Jicama/Yam Bean), 4644 (Malanga), 4672 (Parsnip), 4674 (Peas Green), 4676 (Peas Retailer Assigned), 4723 (Potato Creamer), 4724 (Potato Creamer), 4725 (Potato Russet), 4726 (Potato Long), 4727 (Potato Yellow), 4728 (Potato Retailer Assigned), 4729 (Potato Retailer Assigned), 4730 (Potato Retailer Assigned), 4731 (Potato Retailer Assigned), 4732 (Potato Retailer Assigned), 4733 (Potato Retailer Assigned), 4794 (Taro Root (Dasheen), ), 4795 (Taro Root (Dasheen), ), 4819 (Yuca Root/Cassava/Manioc), 93288 (OrgSwPot/Yam/Kumra-RetAsn), 93321 (Org Celryrt/CelriacW/Leaf), 93474 (Org Sw Potat/Yam/KumraSfn), 94072 (Org Potato Russet), 94074 (Org Sw Potat/Yam/Kumara), 94078 (Org Corn Sweetcorn-Yellow), 94727 (Org Potato Yellow) |
| 7 | Dark green vegetables | 3082 (Broccoli Crowns), 3095 (Kale Multicolor), 3097 (Lettuce Romaine), 3160 (Gai Lan Synon W/ChinBroc), 3163 (Bok Choy-Pak Choi-Shanghi), 3277 (Broccoli Baby), 3322 (Choy Sum/Pak Choi Sum), 3328 (Org Lettuce Mix salad), 3332 (Spinach Baby), 3478 (Quelites), 3479 (Chipilin Leaf), 4060 (Broccoli), 4075 (Lettuce Red Leaf), 4076 (Lettuce Green Leaf), 4090 (Spinach Regular/Bunched), 4543 (Belgian-Endive), 4544 (Bok Choy-Pak Choi-Sm/Bby), 4545 (Bok Choy-Pak Choi), 4547 (Broccoli-Rabe/Gai Lan), 4548 (Broccoli Florettes), 4549 (Broccoli Retailer Assign), 4586 (Chard-SwissChard-Grn), 4587 (Chard-SwissChard-Red), 4588 (Chard-SwissChrd-RetAssgn), 4605 (Escarol/Batavn Chicrygrn), 4614 (Greens Collard), 4616 (Greens Mustard), 4617 (Greens Polk Greens), 4618 (Greens Texas Mustard), 4619 (Greens Turnip), 4620 (Greens Retailer Assigned), 4621 (Greens Retailer Assigned), 4627 (Kale), 4632 (Lettuce Boston/Butter), 4640 (Lettuce Romaine/Cos), 4749 (Spinach Retailer Assignd), 4815 (Watercress), 93095 (Org Kale Multicolor), 93332 (Org Spinach Baby), 94060 (Org Broccoli), 94075 (Org Lettuce Redleaf), 94076 (Org Lettuce Greenleaf), 94090 (Org Spinach Regular/Bunch), 94586 (Org Chard-Swisschd-Grn), 94587 (Org Chard-Swisschd-Red), 94614 (Org Greens Collard), 94615 (Org Greens Dandelion), 94616 (Org Greens Mustard), 94620 (Org Greens RetailAssign), 94627 (Org Kale), 94632 (Org Lettuce Boston/Butter), 94640 (Org Lettuce Romaine/Cos) |
| 8 | Beans, peas, and lentils | 4528 (Beans Fava/Broad), 4529 (Beans Lima), 4532 (Beans Shell), 4535 (Beans Retailer Assigned) |

| **Supplemental Table 2:** Interaction results of baseline child fruit and vegetable intake and fruit and vegetable diversity redeemed (n=1,173)^1^ | | | | | | |
| --- | --- | --- | --- | --- | --- | --- |
|  | Association with change in child dietary intake^2^ | | | | | |
|  | Fruit intake, cups/day | | Vegetable intake^4^, cups/day | | Fruit and vegetable intake^4^, cups/day | |
| **Change in total fruit diversity score^3^** | Interaction β(95%CI) | interaction p-value | Interaction β(95%CI) | Interaction p-value | Interaction β(95%CI) | Interaction p-value |
| Time period 2 vs. Time period 1 | -0.006 (-0.052, 0.040) | 0.803 | 0.003 (-0.031, 0.037) | 0.872 | -0.004 (-0.067,0.060) | 0.914 |
| Time period 3 vs. Time period 1 | 0.0117 (-0.0470,0.0703) | 0.6967 | 0.0403 (-0.0044,0.0850) | 0.0769 | 0.0531 (-0.0230,0.1292) | 0.1715 |
|  |  |  |  |  |  |  |
| **Change in total vegetable diversity score^3^** |  |  |  |  |  |  |
| Time period 2 vs. Time period 1 | -0.0092 (-0.0349,0.0165) | 0.4828 | -0.0139 (-0.0384,0.0107) | 0.2678 | -0.0198 (-0.0581,0.0185) | 0.3110 |
| Time period 3 vs. Time period 1 | 0.0043 (-0.0266,0.0352) | 0.7867 | 0.0047 (-0.0127,0.0221) | 0.5963 | 0.0114 (-0.0235,0.0463) | 0.5224 |
|  |  |  |  |  |  |  |
| **Change in total FV diversity score^3^** |  |  |  |  |  |  |
| Time period 2 vs. Time period 1 | -0.0051 (-0.0183,0.0081) | 0.4466 | -0.0046 (-0.0168,0.0076) | 0.4601 | -0.0084 (-0.0287,0.0118) | 0.4143 |
| Time period 3 vs. Time period 1 | 0.0037 (-0.0139,0.0214) | 0.6808 | 0.0082 (-0.0024,0.0188) | 0.1303 | 0.0129 (-0.0074,0.0332) | 0.2119 |

CVB, cash value benefit; USD, United States dollars; SD, standard deviation; WIC, the Special Supplemental Nutrition Program for Women, Infants, and Children; FV, fruit and vegetables.

^1^Sample size ranges from 727-1,173 due to the number of completed surveys at each timepoint.

^2^Estimate (95% confidence interval) for child dietary intake associations was determined for T2(35USD/month) and T3(24USD/month) compared to T1(9USD/month) in generalized estimating equations linear regression adjusted for child race/ethnicity, sex, and age; household food insecurity and the number of household members under age 18; Models also accommodated clustering of observations within participating children and families, and the interaction between baseline FVI and change in diversity score (e.g. fruit, vegetable, FV).

^3^Change in diversity was calculated as fruit, vegetable, and FV diversity score at time period 2 minus time period 1, and time period 3 minus time period 1, respectively.

^4^Vegetable intake and FV intake included legumes, fried potatoes, and 100% fruit juice.

**Supplemental figure 1**. Fruit (a), vegetable (b), and fruit and vegetable (c) intakes of WIC-participating children in southern California during the augmentation of the CVB
